# Single cell dissection reveals SFRP2+ fibroblasts amplifying inflammatory responses in oral lichen planus

**DOI:** 10.3389/fimmu.2025.1553963

**Published:** 2025-06-12

**Authors:** Juehua Cheng, Jia Liu, Yuchi Zhu, Jingjing Yang, Yanlin Geng, Yuan Fan

**Affiliations:** ^1^ Department of Oral Mucosal Diseases, The Affiliated Stomatological Hospital of Nanjing Medical University, Nanjing, China; ^2^ State Key Laboratory Cultivation Base of Research, Prevention and Treatment for Oral Diseases, Nanjing, China; ^3^ Jiangsu Province Engineering Research Center of Stomatological Translational Medicine, Nanjing, China; ^4^ Department of Oral and Maxillofacial Surgery, The Affiliated Stomatological Hospital of Nanjing Medical University, Nanjing, China

**Keywords:** oral lichen planus, fibroblasts, SFRP2, single-cell sequencing, antigen processing and presenting

## Abstract

**Objectives:**

Oral lichen planus (OLP) is a chronic inflammatory mucosal disease with an incompletely understood pathogenesis. This study aimed to investigate the role of disease-specific fibroblasts in OLP.

**Methods:**

We performed single-cell RNA sequencing on buccal mucosa of 4 OLP patients and one healthy control. Additionally, mRNA expression and immunofluorescence staining were analyzed in primary fibroblasts from 51 OLP patients and 24 healthy individuals. The spatial cellular interactions were assessed using multiplex immunofluorescences in OLP tissues.

**Results:**

Using single-cell RNA sequencing, we identified SFRP2+ fibroblasts as the origin of inflammatory fibroblasts in OLP. A subset of SFRP2+ fibroblasts specifically expressed Wnt5a and was implicated in antigen processing and presentation pathway in OLP. Furthermore, SFRP2+Wnt5a+ fibroblasts amplified and maintained the local immune inflammation by interacting with CD8+ T cells and epithelial cells. Compared to the healthy control group, upregulated expressions of pro-inflammatory molecules (CXCL12, CXCL14) and antigen presenting associated molecules (HLA-A, HLA-B, HLA-C and ERAP2) were displayed in OLP group at mRNA level. Colocalization of SFRP2 and Wnt5a was displayed in the primary cultured fibroblasts of OLP *in vitro*. Besides, SFRP2+ Wnt5a+ fibroblasts were located around CD8+ T cells in the superficial layer of the lymphocyte infiltration zone.

**Conclusions:**

Our findings reveal the heterogeneity and pathogenic mechanisms of fibroblasts in OLP, providing new insights into the cellular drivers of chronic inflammation in OLP.

## Introduction

Oral lichen planus (OLP) is a chronic inflammatory mucosal disease, affecting 1.01% of the global population (1). The main clinical manifestations are bilateral whitish striae accompanied by discomfort and pain. Based on the absence or presence of hyperemia/erosion, patients are classified as erosive oral lichen planus (EOLP) and non-erosive oral lichen planus (NEOLP) (2). The condition of OLP patients is recurrent and prolonged, which affects the daily life of patients. Previous studies have shown the imbalance of regional mucosal immune homeostasis is the core problem leading to the formation of uncontrollable inflammation of OLP, but knowledge about cellular types and interactions involved in OLP is still relatively limited (3).

In recent years, heterogeneous fibroblasts have been reported as dynamic players in autoimmune diseases, including psoriasis, rheumatoid arthritis, and inflammatory bowel disease (4–7). Fibroblasts promote disease initiation, progression, and recurrence by secreting downstream inflammatory factors, presenting antigens, facilitating immune memory, and interacting with immune cells and epithelial cells (8–10). Previous studies have found that fibroblasts secrete CCL5 to regulate the proliferation, migration and apoptosis of CD4+ T cells, but the cellular subtypes and potential mechanisms of pathogenic fibroblasts in OLP remain unclear (11).

Single-cell RNA sequencing (scRNA-seq) is a high-throughput technique for sequencing and analyzing both the genome and transcriptome of individual cells, which generates a view of gene expression over the course of disease development and helps in studying the heterogeneity of disease-specific cell types in immune diseases (12–14). However, few studies were performed in OLP oral mucosal at single-cell level, and the exist studies focused on the contribution of immune cell subpopulation in OLP, such as CD8+ T cells. Recent years, scRNA-seq analysis revealed the significant heterogeneity of fibroblast subsets in several autoimmune diseases, and disease-specific fibroblast subsets can be related to the development and recurrence of autoimmune disease. Thus, finding the knowledge gap of fibroblast heterogeneity and the immune communication network of disease-specific fibroblasts in OLP will contribute to understand OLP pathogenesis.Here, we provide a comprehensive overview of the immunopathogenesis of OLP, based on a single-cell RNA sequencing of oral buccal mucosa from two EOLP patients, two NEOLP patients and one healthy individual, as well as multiple *in vitro* experimental validation. These data further reveal the pathogenesis of OLP, including (1) the characterization of cellular components involved in the pathogenesis of OLP; (2) SFRP2+ fibroblasts involved in the pathogenesis of OLP by production of pro-inflammatory factors; (3) a subset of SFRP2+ fibroblasts contributing to immune disorder through recruiting immune cells and antigen presenting and processing; (4) the interaction among SFRP2+Wnt5a+ fibroblasts, epithelial cells, and CD8+ T cells contributing to amplifying and maintaining the local immune inflammation. Together, our data provide a detailed view of OLP pathology, identifying further mechanisms through which cellular interactions influence inflammatory networks at disease sites, highlighting potential therapeutic targets for interventions, especially fibroblasts.

## Materials and methods

### Clinical samples

Human oral mucosa tissues were collected during a protocol approval from the Ethics Committee of the Affiliated Stomatological Hospital of Nanjing Medical University (PJ2019-281). Each patient gave written informed consent prior to inclusion in the study, and the study was performed in accordance with the Declaration of Helsiniki.

For scRNA-Seq, fresh buccal mucosa samples from 2 non-erosive oral lichen planus (NEOLP) and 2 erosive oral lichen planus (EOLP) were obtained during pathological biopsy, which were confirmed to be oral lichen planus clinically and histopathologically according to the diagnostic criteria proposed by van Meji et al. (15). Patients who had used drugs such as glucocorticoids or immune preparations within 3 months or had used antibiotics within 1 month were excluded from the study. One normal sample was obtained from the buccal mucosa of plastic surgery. Clinical histories were collected (**Supplementary Table S1**; **Supplementary Data 1**).

For immunohistochemistry analysis, KRT17 expression was detected with samples from 20 NEOLP and 23 EOLP. Healthy oral mucosa samples from 18 volunteers treated with plastic surgery were collected, while clinicopathological characteristics of individuals involved in immunohistochemistry are described in **Supplementary Table S2**. Multiplex immunohistochemical staining was performed on 8 OLP samples mentioned above. Fresh tissue samples from some of these patients were collected to isolate the primary fibroblasts for IF and qRT-PCR (**Supplementary Table S3**). All specimens were confirmed histologically by H&E staining.

### Single-cell suspension preparation, cDNA library construction and sequencing

The collected sample tissue was chopped into 0.5mm^3^ block, subjected to enzymatic digestion at 37°C, and passed through a 40μm cell strainer, centrifugated for 5 minutes at 4°C. After the pellet was resuspended with an appropriate amount of medium, added Red Blood Cell Lysis Buffer (MACS, Cat. No. 130-094-183), mixed and stood at 4°C for 10 min, centrifuged for 5 min, and the supernatant was discarded. The pellet was washed, centrifuged at 300g for 5 min, the supernatant was discarded, the cell pellet was resuspended. Adjust the freshly prepared single-cell suspension to 700–1200 cell/μl, and follow the operating instructions (10×Genomics Chromium Next GEM Single Cell 3′ Reagent Kits v3.1, Cat. No. 1000268) for library construction. The constructed libraries were sequenced using the Illumina Nova 6000 PE150 platform.

### Single cell sequencing data analysis

The FASTQ files were processed and aligned to GRCh38 human reference genome using Cell Ranger software (version 9.0.0) from 10x Genomics, with unique molecular identifier (UMI) counts summarized for each barcode. The UMI count matrix was then analyzed using the Seurat R package (version 4.0.0). To filter out low-quality cells and potential multiplet captures, a set of criteria were conducted: (1) gene count < 200, (2) UMI count < 1000, (3) log10GenesPerUMI < 0.7, (4) mitochondrial RNA UMIs > 10%, and (5) hemoglobin RNA UMIs > 5%. Subsequently, the DoubletFinder package (version2.0.3) was used to identify potential doublets. To obtain the normalized gene expression data, library size normalization was processed using the NormalizeData function. After quality control, we identified highly variable genes (HVGs), which are important for distinguishing between cell types. Principal-component analysis (PCA) was performed to reduce the dimensionality with RunPCA function. To correct for batch effects in single-cell RNA-sequencing data, we used the mutual nearest neighbors (MNN) method introduced by Haghverdi et al, implemented with the R package batchelor. Cell visualization was performed using the 2-dimensional Uniform Manifold Approximation and Projection (UMAP) algorithm via the Run UMAP function in Seurat (16). The sequencing and bioinformatics analysis were provided by OE Biotech Co., Ltd. (Shanghai, China).

### Cell clustering and annotation

We used the FindAllMarkers function (test.use = presto) in Seurat to detect marker genes for each cluster, identifying positive markers by comparing a cluster to all other cells. Differentially expressed genes (DEGs) were selected using the function FindMarkers (test.use = presto). P value < 0.05 and |log2foldchange| > 0.58 was set as the threshold for significantly differential expression. Additionally, the R package SingleR (version 1.4.1) was applied to independently infer the origin and classify the cell types of each single cell.

### Cell trajectory analysis

We conducted cell trajectory analysis using the Monocle2 package. First, the raw counts were converted from the *Seurat* object to a CellDataSet object using the importCDS function in Monocle. To select ordering genes likely informative for cell ordering along the pseudotime trajectory, the differentialGeneTest function (qval < 0.01) was applied. Dimensionality reduction clustering was carried out with the reduceDimension function, followed by trajectory inference using orderCells with default settings. Changes in gene expression over pseudotime were visualized with the plot_genes_in_pseudotime function (17).

### Gene Set Variation Analysis

To perform the Gene Set Variation Analysis, the GSEABase package (version 1.44.0) was used to load the gene set file which was downloaded and processed from KEGG database (https://www.kegg.jp/). Pathway activity scores for individual cells were assigned using GSVA (version 1.30.0) with default settings. Differences in pathway activity between cells were analyzed using the LIMMA package (version 3.38.3).

### Cell communication

The CellPhoneDB (v2.0) was used to identify biologically relevant ligand-receptor interactions. A ligand or receptor was considered “expressed” in a cell type if at least 10% of the cells in that type had non-zero read counts for the corresponding gene. Statistical significance was determined by shuffling cluster labels and repeating the analysis to generate a null distribution for each ligand-receptor (LR) pair across cell type comparisons (18). After 1,000 permutations, P-values were calculated based on the normal distribution of permuted interaction scores. Cell-cell communication networks were defined by linking two cell types where the ligand was expressed in one and the receptor in the other. R packages Igraph (version1.2.4.1) and Circlize (version 0.4.8) were used to display the cell-cell communication networks.

### SCENIC analysis

The SCENIC analysis (version1.2.4) was run using the motifs database for RcisTarget and GRNboost with the default parameters. The RcisTarget package (version1.10.0) was used to identify transcription factor (TF) binding motifs that were over-represented in the gene list. The AUCell package (version1.12.0) was subsequently employed to score the activity of each regulon group within individual cells.

### Isolation and culturing of primary fibroblasts

Fresh tissues were washed in PBS containing 5% penicillin-streptomycin-glutamine three times, minced into fragments and digested with collagenase type I (2g/ml) for 30 min. Primary fibroblasts were cultured in DMEM containing 10% FBS and 1% penicillin-streptomycin at 37 °C in a 5% CO2 incubator. The morphology of the primary cultured fibroblasts is shown in **Supplementary Figure S2A** (**Supplementary Data 2**). Identification of primary cultured fibroblasts was performed in **Supplementary Figures S3A, B** by immunofluorescence. Primary cultured fibroblasts were used between passage 3-6.

### Immunofluorescence

Cells (1×10^5^) were seeded on cover slips and cultured overnight or until reaching 70% confluency. For immunofluorescence, the cells were fixed, permeabilized, and blocked with 3% bovine serum albumin for 30 minutes at room temperature, followed by overnight incubation with primary antibodies at 4°C. Afterward, cells were incubated with secondary antibodies for 60 minutes at room temperature in the dark. Nuclei were counterstained with DAPI (Beyotime, Shanghai, China) for 5 minutes, and the cover slips were mounted and imaged using a fluorescent microscope (Leica Microsystems, Germany). The antibodies used are listed in **Supplementary Table S4**.

### RNA isolation and RT-qPCR

Total RNA was isolated from primary cultured fibroblasts using the MolPure Cell RNA Kit (Yeasen Biotechnology, Shanghai, China) and reverse transcribed with PrimeScript™ RT Master Mix kit (Takara, Shiga, Japan). was performed qPCR with a LightCycler96 (Roche, Basel, Switzerland) using SYBR Green™ Master Mix (Takara, Shiga, Japan). The results were normalized to ACTB expression level, and the primer pairs used are listed in **Supplementary Table S5**.

### H&E staining and immunohistochemistry

For H&E staining and IHC, 4μm formalin-fixed mucosa sections were dewaxed and rehydrated firstly. For IHC, the slides underwent antigen retrieval in citrate buffer at 100°C for 20 minutes. Afterward, they were incubated with primary antibodies at 37°C for 2 hours, followed by a 30-minute incubation with HRP-conjugated secondary antibodies. Finally, the sections were stained with DAB solution, counterstained with a neutral background reagent, and observed under a microscope. Image analysis software was used for detailed section analysis and to obtain the IHC score. The antibodies used are shown in **Supplementary Table S4**.

### Multiplex immunohistochemistry

Multiplex immunohistochemistry (mIHC) staining was carried out using the tyramide signal amplification (TSA) 4-color IHC kit (Panovue, Beijing, China) following the manufacturer’s protocol. Sections of 4μm thickness from paraffin-embedded samples were first dewaxed, rehydrated, and then subjected to antigen retrieval at 100°C. Subsequently, the sections were incubated overnight at 4°C with the first primary antibody, followed by a 30-minute incubation with HRP-conjugated secondary antibodies, and a 15-minute incubation with TSA. After washing with TBST buffer, the slides underwent antigen retrieval in citrate buffer for 20 minutes. This process was repeated for the next primary antibodies. Each slide was then treated with Antifade Mounting Medium containing DAPI and manually coverslipped. Images were captured using the Olympus soft imaging system. The antibodies and reagents used are detailed in **Supplementary Table S4**.

### Statistical analysis

The scRNA-seq data was analyzed by R package SingleR, differentially expressed genes were identified using the Seurat package, and hypergeometric test was used for the enrichment analysis. The Data of IHC and qPCR were analyzed on GraphPad Prism software version 10.1.1 and presented as the mean ± standard deviation (SD). The results between two groups were analyzed using the two-tailed unpaired Student’s t-test or Mann Whitney test (*P<0.05, ** *P*<0.01, *** *P*<0.001, **** *P*<0.0001).

## Results

### scRNA-seq profiling of the cell composition from healthy control, NEOLP and EOLP

**Figure 1:**
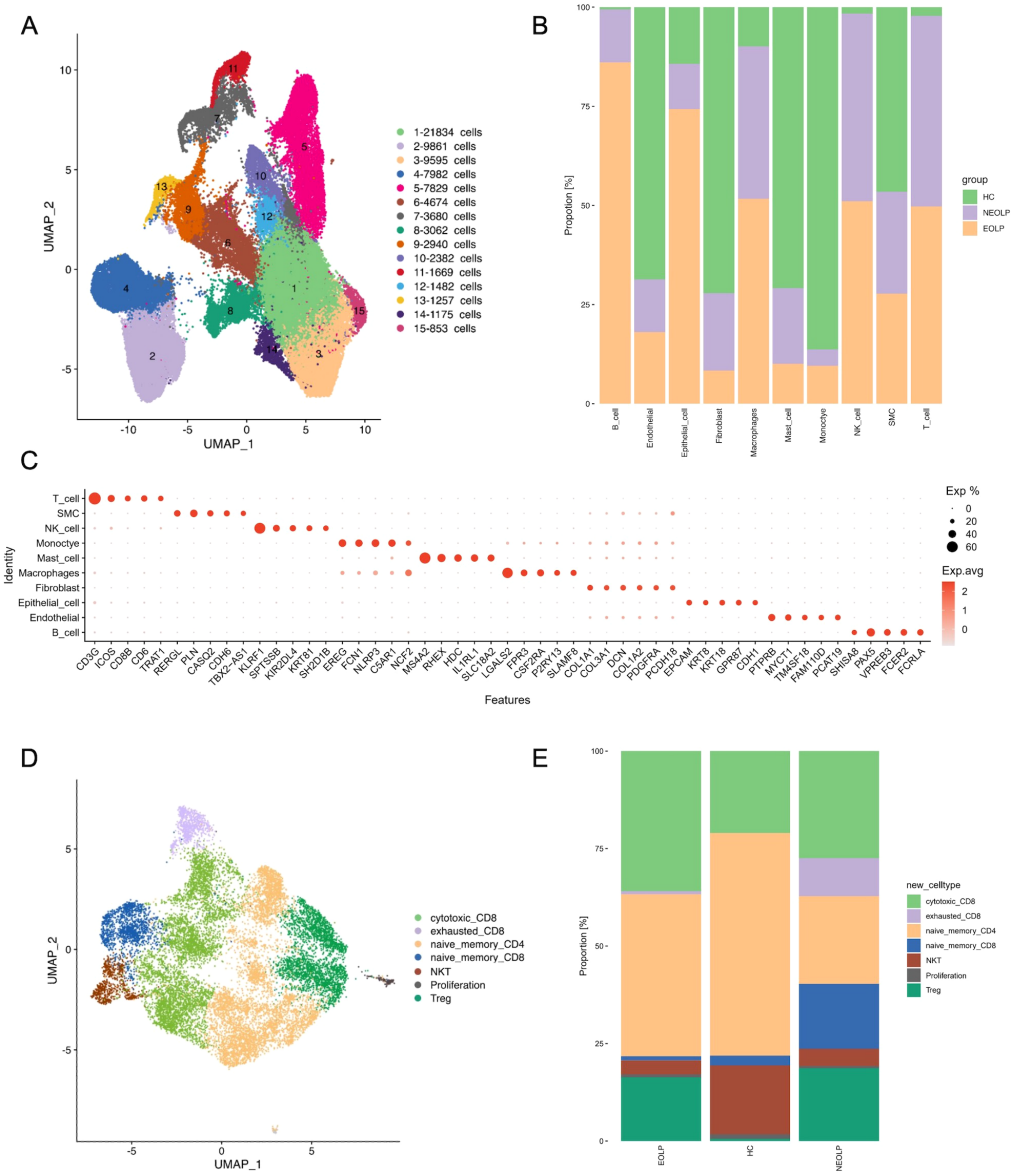
scRNA-seq profiling of the cell composition from healthy control, NEOLP and EOLP tissue samples. **(A)** The uniform manifold approximation and projection (UMAP) of the 76,657 cells profiled here showing cells colored by 15 sub-populations. **(B)** Bar plot showing the 10 cell types colored by disease conditions, originating from HC, NEOLP and EOLP group. **(C)** Dot plot analysis showing representative marker genes for each cell type. The color scale represents the scaled expression of each gene. **(D)** UMAP plot displaying cells categorized by T cell types. **(E)** Bar plot showing the different compositions of T cell types across HC, NEOLP and EOLP group.

To gain insight into the unbiased cellular composition and comprehensive cell states of OLP, we chose buccal mucosa biopsies from 1 healthy control, 2 NEOLP (non-erosive oral lichen planus) patients and 2 EOLP (erosive oral lichen planus) patients. A total of 76,657 high-quality single cells were profiled using the 10X Genomics Chromium Droplet platform. We selected variable genes and performed UMAP dimensionality reduction, which led to the identification of 15 sub-populations (**Figure 1A**). We calculated marker genes and annotated 10 primary cell types including T cells (CD3G, ICOS, CD8B, CD6), smooth muscle cells (SMC; PLN, RERGL, CDH6), NK cells(KLRF1, SPTSSB, NKG7), monocyte (EREG, FCN1, NLRP3, C5AR1), mast cells (MS4A2, RHEX, HDC), macrophages (LGALS2, FPR3, CSF2RA), fibroblasts (COL1A1, COL1A2, DCN, COL3A1, PDGFRA), epithelial cells (KRT8, KRT18, CDH1, EPCAM), endothelial cells (PECAM1, CDH5, VWF) and B cells (CD19, CD79A, MS4A1) (**Figures 1B, C**). The identified cell types included cells from the majority of HC, NEOLP, and EOLP libraries, indicating that each cell type was linked to a common lineage rather than originating from a single donor (**Figure 1B**, **Supplementary Figure S1A**).

Analysis of the cell type composition revealed an increased proportion of T cell in both NEOLP and EOLP lesions, identifying T cells as the major hallmark cell type of OLP (**Figure 1B**). However, no significant differences in cell composition were found between NEOLP and EOLP. Additionally, macrophages and NK cells were elevated in both NEOLP and EOLP, while monocytes were decreased compared to HC.

To define the roles of T cell subclusters in OLP, we sub-clustered the T cells and identified 7 cell subtypes in NEOLP, EOLP and HC: cytotoxic CD8+ T cells (IL2, GZMA, GNLY), exhausted CD8+ cells (LAG3, TIGIT, PDCD1), naïve memory CD4+ T cells (CD4, TCF7, SELL, LEF1), naïve memory CD8+ T cells (TCF7, SELL, LEF1, GZMK), NKT cells (KLRD1, KLRF1,TRDC), proliferation (MKI67, CDK1, STMN1), Treg (IL2RA, FOXP3, IKZF2) (**Figure 1D**). Compared to HC, the proportion of cytotoxic CD8+T cells and Treg increased in NEOLP and EOLP (**Supplementary Figure S1B, C**). Given that cytotoxic CD8+ T cells are thought to be the main lymphocytes in OLP, the KEGG analysis of top 20 differentially expressed genes in NEOLP and EOLP cytotoxic CD8+ T cells revealed enrichment in antigen processing and presentation, IL-17 signaling pathway and Th17 cell differentiation compared with healthy control (**Supplementary Figure S1D, E**). Our data also revealed that NEOLP had a higher proportion of naïve memory CD8+ T cells and exhausted CD8+T cells compared to EOLP (**Figure 1E**). The KEGG analysis of top 20 differentially expressed genes in exhausted CD8+T cells from NEOLP to EOLP stages indicated an enrichment in antigen processing and presentation, hematopoietic cell lineage, IL-17 signaling pathway and Th17 cell differentiation (**Supplementary Figure S1F**).

### Fibroblasts involved in immunity disorder of OLP by secreting pro-inflammatory factors

**Figure 2:**
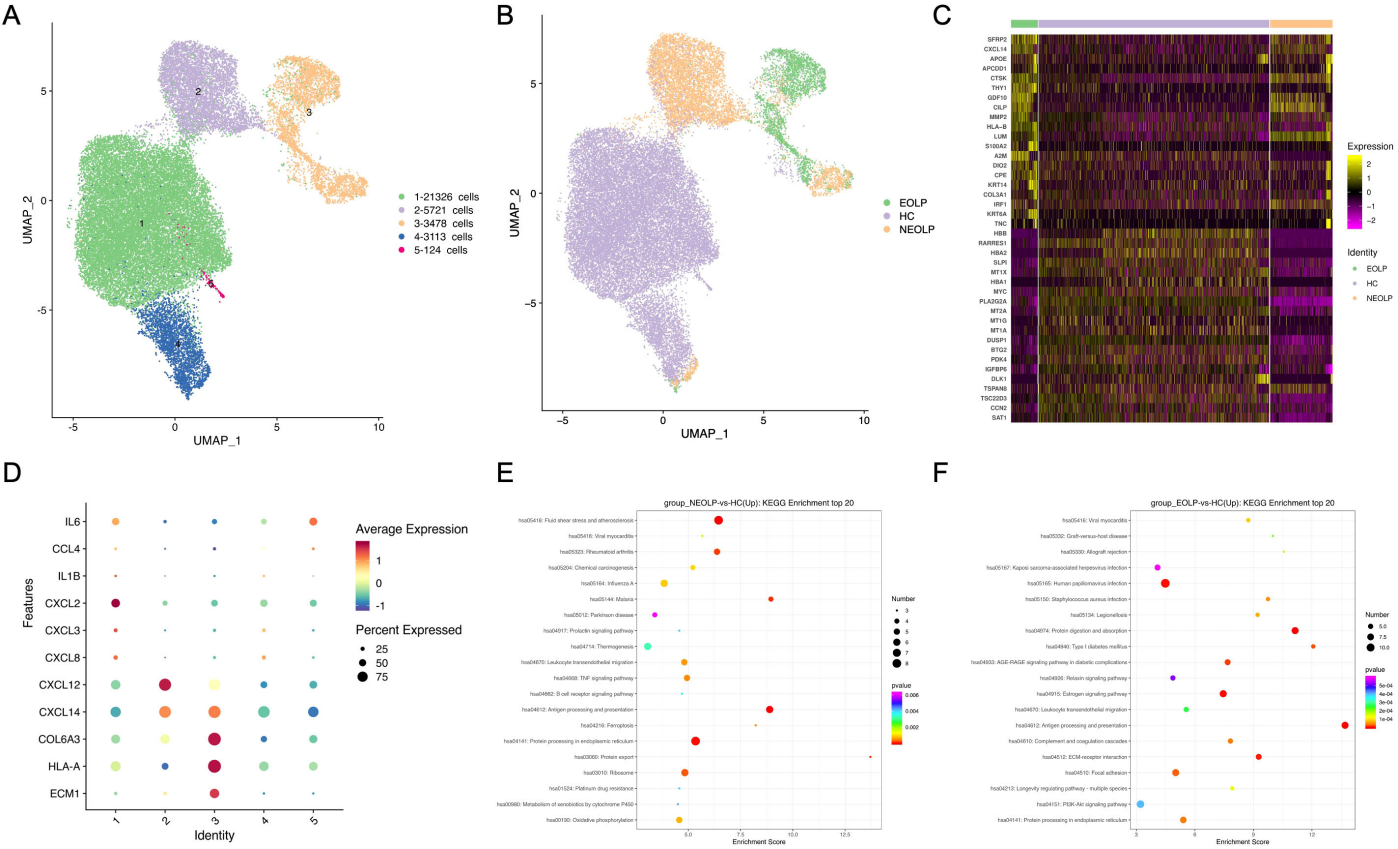
Fibroblasts involved in immunity disorder of OLP by secreting pro-inflammatory factors. **(A)** UMAP plot showing 33,762 fibroblasts colored by 5 sub-clusters. **(B)** UMAP plot showing fibroblasts colored by diverse disease conditions. **(C)** Compared to HC group, heatmap showing the top 20 up-regulated and down-regulated genes in NEOLP and EOLP. **(D)** Dot plot analysis of highly expressed pro-inflammatory factors across 5 subclusters of fibroblasts types. The color scale represents the scaled expression of each gene. **(E, F)**. Dot plot of KEGG enrichment analysis showing the DEGs of fibroblasts between NEOLP and HC, EOLP and HC.

Since fibroblasts were the second most abundant cell type in OLP lesions in our data, we explored their contribution to the disease. We further sub-clustered all fibroblasts, a total of 33,762 cells into 5 sub-clusters based on previously annotated expression markers (**Figure 2A**, **Supplementary Figure S2A**). Notably, fibroblasts in NEOLP and EOLP were mainly consisted of clusters 2 and cluster3 (**Figure 2B**, **Supplementary Figure S2B**). To define the genes contributed with OLP, we performed differential expression analysis for the top 20 up-regulated genes in disease states. Significantly, SFRP2 was highly ranked among the up-regulated genes in NEOLP and EOLP (**Figure 2C**). Moreover, the expression of CXCL14, APOE, CTSK, THY1, MMP2, A2M, TNC had shown an increase in NEOLP and EOLP top 20 up-regulated genes. We examined across subclusters the expression of pro-inflammatory factors that previously reported to be involved in OLP pathogenesis by subclusters, and found CXCL14, COL6A3, extracellular matrix protein 1 (ECM1), HLA-A genes were highly expressed in clusters 3, which was mainly composed of EOLP (**Figure 2D**). And we found CXCL12 and CXCL14 genes were highly expressed in clusters 2, which was mainly composed of NEOLP. Consistently, increased mRNA expression of chemokines (CXCL14, CXCL12, CCL19) was found in primary fibroblasts from NEOLP and EOLP tissue samples (**Supplementary Figures S4A-4D**).

KEGG analysis of DEGs with increased expression from NEOLP and EOLP indicated enrichment in antigen processing and presentation pathways (**Figures 2E, F**). Compared to HC, fibroblasts in NEOLP were enriched in the protein processing endoplasmic reticulum pathway, while fibroblasts in EOLP showed enrichment in ECM-receptor interaction pathway. Based on the pathogenic fibroblast markers in previous autoimmune diseases, we detected the expression of related pathogenic fibroblast markers in the three groups (**Supplementary Figure S2C**, **Supplementary Data 3**) (19–22). Our fibroblasts in NEOLP and EOLP shared transcriptional similarity with fibroblasts from skins of scleroderma patients (including CCL19+APOE+CXCL12+ fibroblasts and SFRP2+PRSS23+ fibroblasts), which indicated an overlap between inflammatory features of fibroblasts across OLP and other diseases. Collectively, these results highlight the heterogeneity among fibroblasts, which contribute to the production of extracellular matrix components, as well as to pro-inflammatory compartments and cytokine production.

### SFRP2+ Wnt5a+ fibroblasts were specific contributor of OLP immunological responses

**Figure 3:**
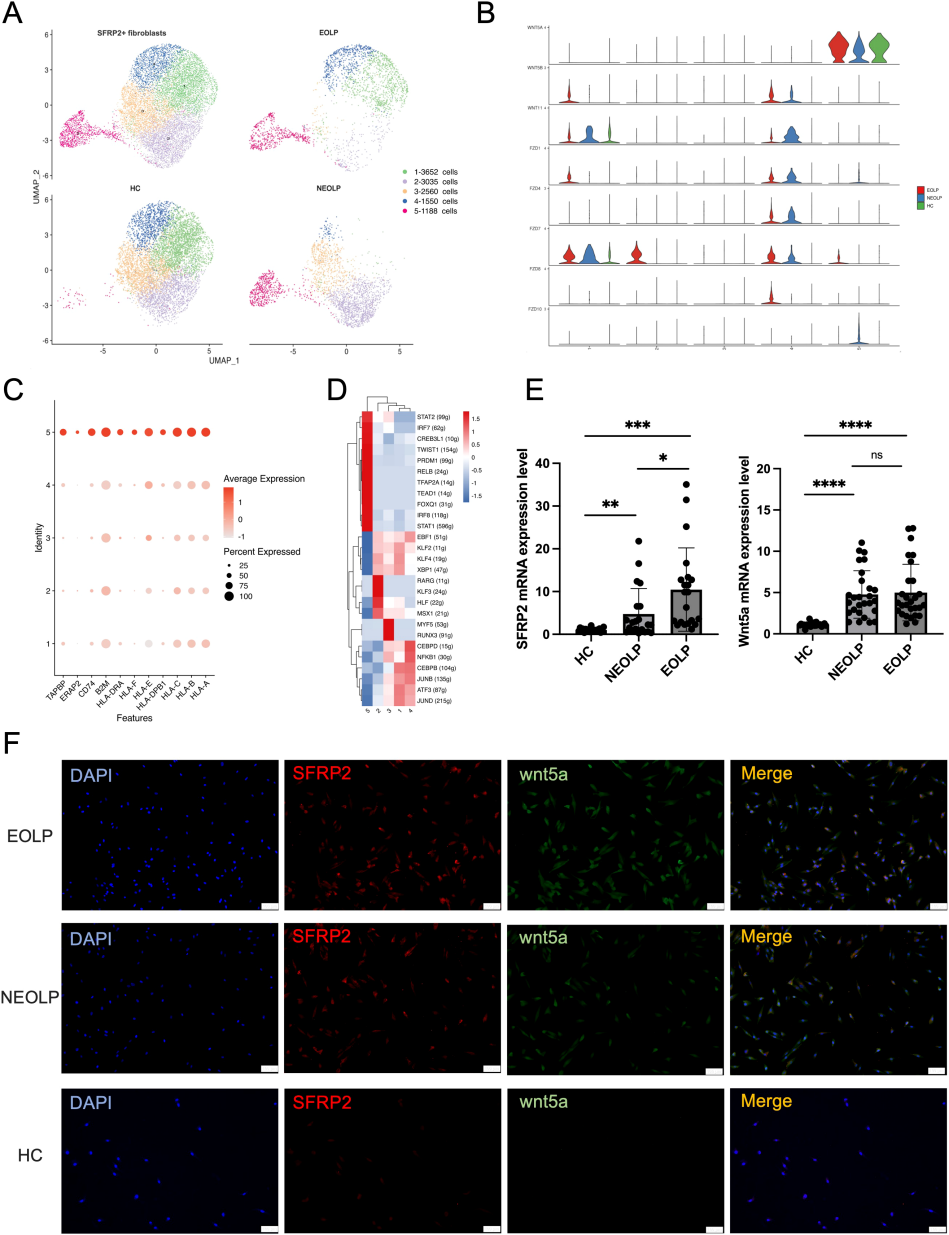
SFRP2+ Wnt5a+ fibroblasts were specific contributor of OLP immunological responses. **(A)** UMAP plot showing SFRP2+ fibroblasts colored into 5 differential cell types, and colored by diverse disease conditions. **(B)** Violin plot showing the expression of gene split by subcluster of SFRP2+ fibroblasts. **(C)** Dot plot showing the expression of antigen presenting associated genes in each subcluster of SFRP2+ fibroblasts. The color scale represents the scaled expression of each gene. **(D)** Heatmap showing up-regulated regulon activity across 5 different SFRP2+ fibroblasts subtypes. **(E)** mRNA expression of SFRP2 and Wnt5a in primary cultured fibroblasts. *p< 0.05, *p< 0.01, ***p< 0.001, ****p< 0. 0001, ns, not significant. (HC, n=24; NEOLP, n=25; EOLP, n=26) **(F)** Immunofluorescence of primary cultured fibroblasts, SFRP2 (red), Wnt5a (green), Merge (yellow). Scale bar:100μm. **p<0.01.

As SFRP2 was the top up-regulated gene in OLP fibroblasts compared HC, we investigated the contribution of SFRP2+ fibroblasts to the disease. We sub-clustered SFRP2+ fibroblasts into 5 subclusters (**Figure 3A**, **Supplementary Figure S5A**). Within the SFRP2+ fibroblasts, the sub-cluster 5 was primarily composed of NEOLP and EOLP, while the sub-cluster 1–4 included cells from all three groups (**Figure 3A**). KEGG analysis of DEGs with increased expression of SFRP2+ fibroblasts from NEOLP and EOLP indicated enrichment in antigen processing and presentation pathways (**Supplementary Figures S6A, B**).

Interestingly, the sub-cluster 5 of SFRP2+ fibroblasts specially expressed Wnt5a, which was detected as a highly ranked ligand-receptor pair in communication with T cells in OLP (**Figure 3B**, **Supplementary Figure S5B**). Furthermore, we examined the expression of antigen presenting associated genes, and found a greatest increase of with antigen presenting associated genes, including HLA-A, HLA-B, HLA-C, HLA-E, HLA-DRA, APOE and ERAP2, TAPBP (**Figure 3C**). While compare with HC, the mRNA expression of HLA-A, HLA-B, HLA-C, ERAP2, CD74, APOE were detected an increase in primary cultured fibroblasts of NEOLP and EOLP (**Supplementary Figures S4E-4J**).

Combined with transcription factors regulon activity score and ranking plot, sub-cluster 5 of SFRP2+ fibroblasts enriched in signal transducer and activator of transcription (STAT1, STAT2), interferon regulatory factors (IRF7, IRF8), the twist-related protein 1 (TWIST1), the absence of Blimp-1 (PRDM1), NF-kappa B family transcription factors (RELB), transcription factor AP-2-α (TFAP2A), TEA domain family gene (TEAD1), and the forkhead box protein family gene (FOXQ1) (**Figure 3D**). Compared to HC, the increased mRNA expression of SFRP2 and Wnt5a was found in primary fibroblasts of NEOLP and EOLP samples (**Figure 3E**). Further, immunofluorescence showed the increased expression and the colocalization of SFRP2 and Wnt5a in primary cultured fibroblasts of NEOLP and EOLP (**Figure 3F**). Taken together, SFRP2+ Wnt5a+ fibroblasts may involve in processing and presentation pathway of OLP.

### Distinct SFRP2+ fibroblasts states reflect immunology responses in OLP

**Figure 4:**
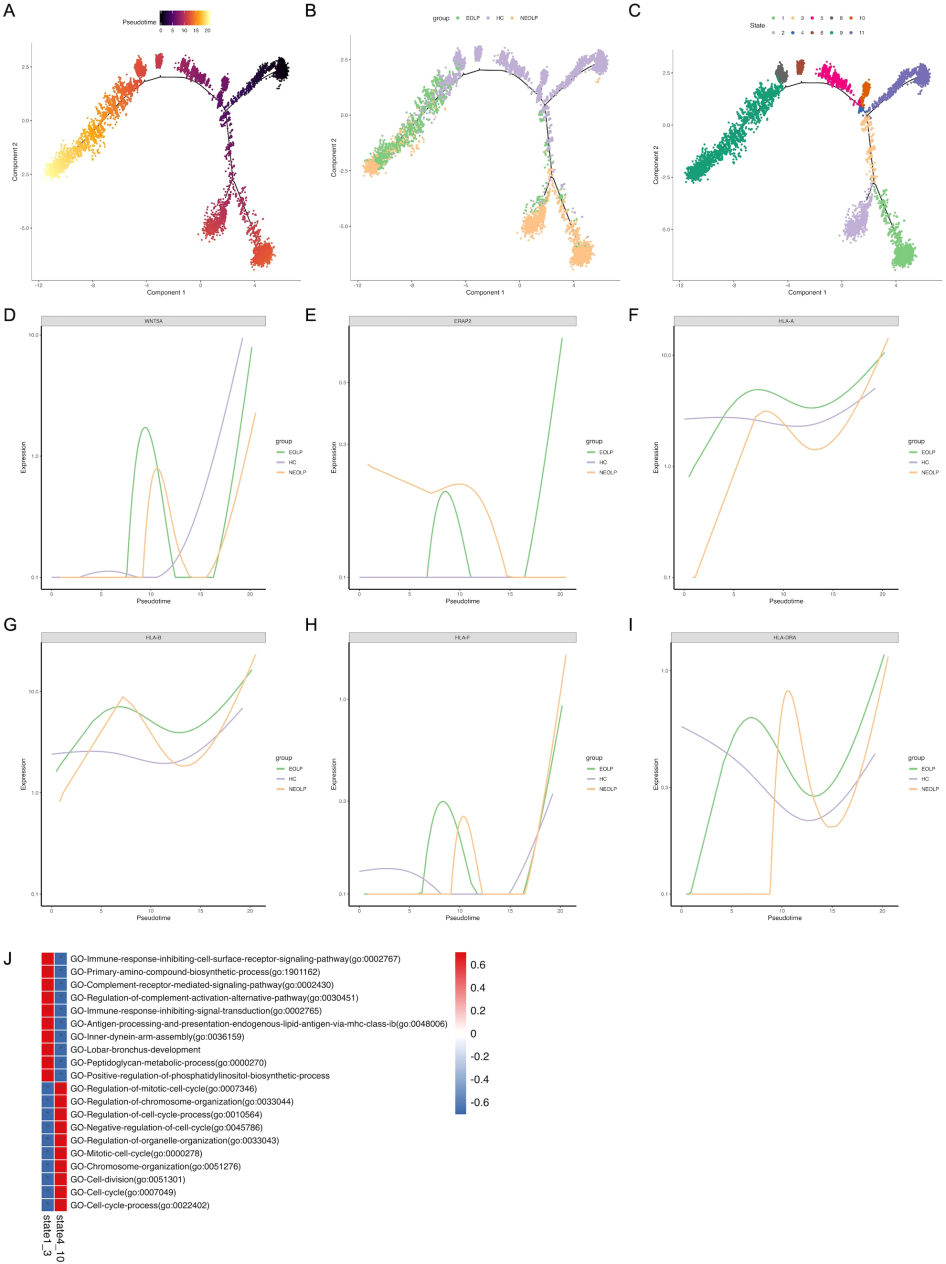
Distinct SFRP2+ fibroblasts states reflect immunology responses in OLP. **(A. B)** Pseudotime trajectory of SFRP2+ fibroblasts arranged along the disease state of OLP **(A)** and the chronological order **(B)**. **(C)** Pseudotime trajectory of SFRP2+ fibroblasts annotated diverse cell types along chronological order. **(D-I)** Pseudotime gene expression of antigen-presenting molecules in SFRP2+ fibroblasts along the chronological order. **(J)** GO analysis of DEGs in SFRP2+ fibroblasts.

To further investigate the relationship between SFRP2+ fibroblasts from healthy control and OLP, we conducted monocle pseudotime analysis, which arranged SFRP2+ fibroblasts along a linear trajectory. SFRP2+Fibroblasts were divided into 11 subpopulations in chronological order (**Figures 4A–C**). Interestingly, we found that SFRP2+ fibroblasts followed a progression of HC to NEOLP and EOLP. Subgroup 11 of SFRP2+ fibroblasts consisted primarily of cells from HC, while the remaining subpopulations split into two main branches: group 1–3 and group 4-10, based on a time bifurcation point.

To verify the potential pro-inflammation cytokines driving differentiation, we analyzed variable genes along the pseudotime trajectory. We found that Wnt5a module scores were positively correlated with fibroblasts pseudo time, while modules scores for antigen-presenting molecules, including ERAP2, HLA, HLA-B, HLA-F and HLA-DRA, were highly correlated with OLP SFRP2+ fibroblasts pseudo time. This was consistent with the results inferred from differential genes in fibroblasts across each disease states (**Figures 4D–I**).

The gene ontology (GO) analysis of differently expressed genes (DEGs) in state1–3 indicated a gradual enrichment of antigen processing and presentation endogenous lipid antigen via MHC class I, immune response inhibiting cell surface receptor signaling pathway, complement receptor mediated signaling pathway, immune response inhibiting signal transduction (**Figure 4J**). These results suggest that SFRP2+ fibroblasts in OLP are associated with antigen presentation and inhibition of cell proliferation, and may be related to inhibition of basal cell proliferation and antigen presentation of immune cells.

### Phenotypic changes of keratinocytes with different differentiation states under inflammatory conditions in OLP

**Figure 5:**
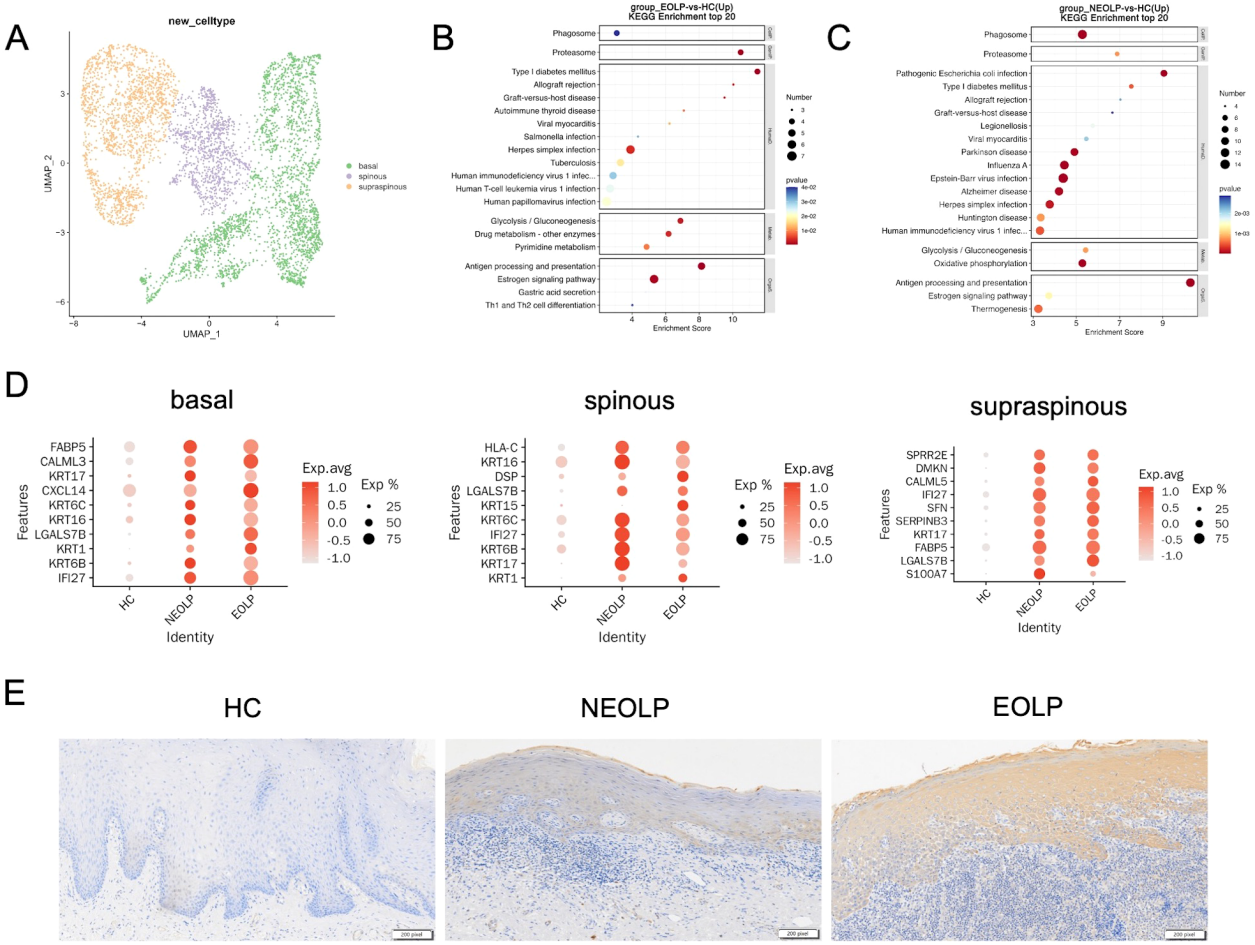
Phenotypic changes of keratinocytes with different differentiation states under inflammatory conditions in OLP. **(A)** UMAP plot showing epithelial cells colored by 3 cell types. **(B, C)** Dot plot of KEGG enrichment analysis of DEGs between NEOLP and HC, EOLP and HC in epithelial cells. **(D)** Dot plot analysis of top 10 differential expressed genes comparing HC to NEOLP and EOLP in the basal (left), spinous (middle), and supraspinous (right) layers. The color scale represents the scaled expression of each gene. **(E)** Immunohistochemistry showing the expression of cytokeratin17 in the epithelial layer of HC, NEOLP and EOLP tissue. Scale bar:200 pixels.

Considering the current understanding of pathological features of epithelial layer in OLP, we further characterized epithelial cells to explore their potential functions. We subclustered epithelial cells and annotated the isoforms by three differentiation status: basal lamina (COL17A1, DST, KRT15), spinous lamina (KRT6A, KRT6B, KRT16), and supraspinous lamina (SLURP1, KLK7, KRT2) (**Figure 5A**, **Supplementary Figures S7A-C**). We ran differential gene expression analysis of keratinocyte with differentiation status (basal, spinous, supraspinous) in NEOLP and EOLP, compared to HC. Interestingly, KRT17 (a potential antigen related gene), IFI27 (type I interferon-induced genes) and LGALS7B (an apoptosis-related gene) were highly ranked in the top 10 up-regulated gene of NEOLP and EOLP at three differentiation status (**Figure 5D**).

We then examined the protein expression of KRT17 in oral mucosal tissues by immunohistochemistry and the results showed that KRT17 was prominently expressed in the keratinocytes of NEOLP and EOLP, with higher expression in EOLP compared to NEOLP (**Figure 5E**, **Supplementary Figure S7H**).

Furthermore, KEGG analysis of up-regulated DEGs from NEOLP and EOLP indicated enrichment of antigen processing and presentation pathways in both basal and spinous layers (**Figures 5B, C**, **Supplementary Figure S7E**). Other upstream inflammatory responses in the basal layer of NEOLP and EOLP mucosa included IL17 signaling pathway (**Supplementary Figure S7D**). Th1 and Th2 cell differentiation pathway, Th17 cell differentiation pathway and cell adhesion molecules pathway were upregulated in the spinous layer of NEOLP and EOLP mucosa (**Supplementary Figure S7E**). Compare to HC, the oxidative phosphorylation signaling pathway and thermogenesis was predominately enriched in the supraspinous layer of NEOLP and EOLP (**Supplementary Figure S7F**). In addition, the results of KEGG analysis suggested an active pro-inflammatory environment potentially driving disease progression.

### Cell-to-cell communication analysis of specific network among SFRP2+ fibroblasts, CD8+ T cells and epithelial cells in OLP

To assess changes that occur in oral lichen planus, we further analyzed how the cell-to cell communication changes in the development of the disease. Firstly, we aggregated the strength scores of interred interactions, and found that the interaction scores increased from HC to NEOLP and EOLP (**Supplementary Figure S8A**). Surprisingly, fibroblasts communicated closely with many cell types through putative ligand-receptor interactions in NEOLP and EOLP (**Supplementary Figure S8B**). Previous researchers have also found that fibroblasts in OLP communicate closely with other cell types, which is consistent with the results of this study.

We then analyzed the ligand-receptor pairs changes involving fibroblasts in NEOLP and EOLP. Notably, fibroblasts were the source of CXCL12 and interacted with the receptors of CXCR4 on T cells and NK cells, suggesting an important role of fibroblasts in recruiting immune cells in oral lichen planus (**Supplementary Figure S8C**). In addition, fibroblasts also expressed fibroblast growth factor (FGF2, FGF7, FGF10), which interacted with FGFR1 in endothelial cells by activating pro-angiogenic activity.

Since epithelial cells and T cells play a major role in the pathogenic mechanisms currently known for OLP, and given the observed shifts in cell type of T cells and epithelial cells with specific differential states, we further performed the ligand-receptor analyses on the specific subcluster of SFRP2+ fibroblasts (subcluster1-5), epithelial cells (basal, spinous, supraspinous) and T cells (cytotoxic CD8+ T cells, exhausted CD8+ T cells, naïve CD4+ T cells, naïve CD8+ T cells, Treg).

**Figure 6:**
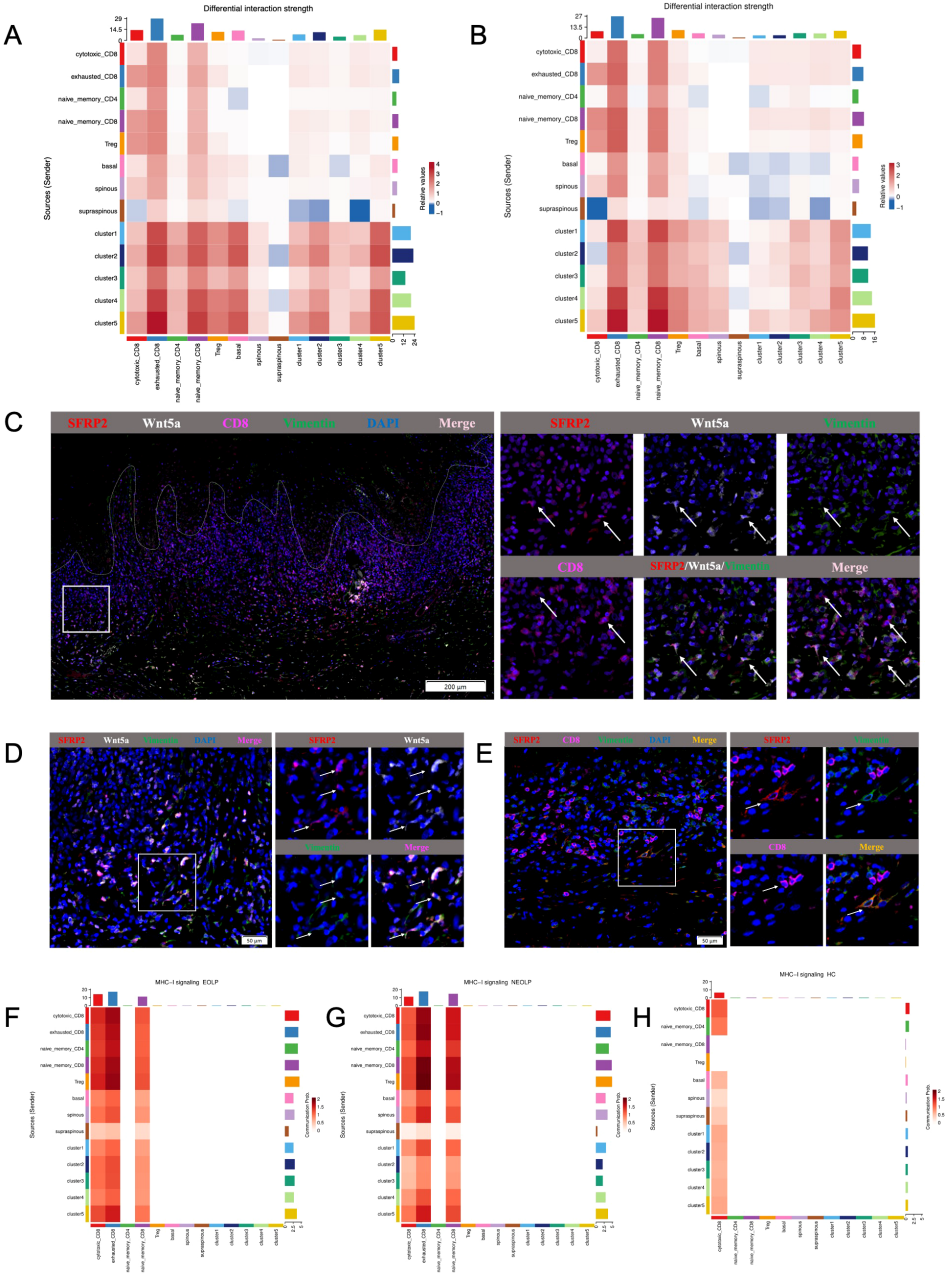
Cell-to-cell communication analysis of specific network among SFRP2+ fibroblasts, CD8+ T cells and epithelial cells in OLP. **(A, B)** Heatmap showing the differential interaction strength between the NEOLP and HC, EOLP and HC of the 5 subclusters of SFRP2+ fibroblasts, subclusters of epithelial cells (basal, spinous and supraspinous) and subclusters of T cells. **(C)** The mIHC staining of SFRP2 (red), Wnt5a (white), CD8 (pink) and Vimentin (green) in the samples of OLP buccal mucosa. Scale bars= 200μm. **(D)** The mIHC staining of SFRP2 (red), Wnt5a (white) and Vimentin (green)in the samples of OLP buccal mucosa. Scale bars=50μm. **(E)** The mIHC staining of SFRP2 (red), CD8 (pink) and Vimentin (green) in the samples of OLP buccal mucosa. Scale bars=50μm. **(F-H)**. Heatmap showing the MHC-I signaling interaction strength between T cells, fibroblasts and epithelial cells at NEOLP, EOLP and HC stages.

The greatest number of ligand-receptor pair changes in NEOLP and EOLP was observed among three cell types: the cluster5 of SFRP2+ fibroblasts, exhausted CD8+ T cells and naïve CD8+ T cells (**Figures 6A, B**). Interestingly, we found that the cluster5 of SFRP2+ fibroblasts connected closely with cytotoxic CD8+ T cells, exhausted CD8+ T cells and naïve CD8+ T cells by ligand-receptor pair involving the MHC-I signaling pathway (**Figures 6F-H**, **Supplementary Figure S8D**). In return, cytotoxic CD8+ T cells and exhausted CD8+ T cells expressed cytotoxic genes (IFNG and GZMA), that further connected with the cluster4 and cluster5 of SFRP2+ fibroblasts with interferon gamma receptors (IFNGR1, IFNGR2) and thrombin-activated factor 2 receptor (F2R) (**Supplementary Figures S9A, B**). Cytotoxic CD8+ T cells also expressed vascular cell adhesion molecule 1 (VCAM1) which connected with the cluster5 of SFRP2+ fibroblasts in the integrins (ITGA4, ITGB1, ITGB7) (**Supplementary Figure S9D**). In addition, SFRP2+Wnt5a+ fibroblasts were expressed around CD8+ T cells in the superficial layer of the lymphocyte infiltration zone and communicated closely with CD8+ T cells, as reveled by multiple immunofluorescence (**Figures 6C-E**). Taken together, these results reveal that SFRP2+ fibroblasts contribute to inflammatory cell infiltration and are in close proximity to CD8+ T cells in the superficial layer of the lymphocyte infiltration zone in oral lichen planus.

In epithelial cells of NEOLP and EOLP, the greatest number of ligand-receptor pair changes was showed in the basal lamina. SFRP2+ fibroblasts expressed tenascin family members (TNC, TNXB), which are known as chronic inflammation related genes, linking to receptors expressed by the epithelial cells of basal layer (**Supplementary Figure S9C**). Compare with HC, the mRNA expression of TNC were detected an increased expression in primary cultured fibroblasts of NEOLP and EOLP (**Supplementary Figure S4K**). TNC has the capacity to enhance the epithelial-mesenchymal transition (EMT) of keratinocytes, suggesting a potential role for fibroblasts in the epithelial-mesenchymal transition (EMT) alteration of OLP epithelial cells.

## Discussion

Herein, our work detailed that SFRP2+ fibroblasts contribute to local immune inflammation of OLP within a transition into pro-inflammatory state directly, and promote the local immune network indirectly via crosstalk among CD8+ T cells and epithelial cells, which helps to reveal the potential function of heterogeneous fibroblasts in OLP and better understand unique cellular contributors involved in OLP pathological mechanisms.

Fibroblasts are known to contribute to the formation and maintenance of extracellular matrix components; however, the potential role of pathogenic fibroblasts in autoimmune diseases has been reported based on the computational inference of intercellular interactions using single-cell sequencing data in recent years (9, 23–26). On the one hand, fibroblasts aggravate the local immune inflammatory response by directly secreting inflammatory factors (27). On the other hand, fibroblasts mediate the recruitment of immune cells and differentiation of immune progenitor cells by secreting chemokines (5, 28–30). Reports indicated that CCL19+ fibroblasts and IFN-activated fibroblasts drive the T cell recruitment, a shared feature of inflammation across multiple diseases (31). Additionally, ECM1 was reported to stimulate fibroblast-to-myofibroblast transition and up-regulates inflammatory pathways in irreversible fibrosis (32). Here, our data indicated that fibroblasts amplified local inflammatory disorder via secreting pro-inflammatory molecules (CXCL14, CXCL12, CCL19, MMP2, ECM1, COL6A3) in OLP. Consistent with previous reports, our data also provided that fibroblasts contribute to infiltration of CCR4+ T cells in local immune environment via production of CXCL12 in OLP (33–35). This suggests that fibroblasts mediate local inflammation via production of pro-inflammatory molecules and recruiting immune cells in OLP.

Further, we identified for the first time that a subset of SFRP2+ fibroblasts in OLP specifically expressed Wnt5a, and was the leading player in the pro-inflammatory subset of OLP. Emerging evidence highlights the pleiotropic role of SFRP2+ fibroblasts in orchestrating immune regulatory networks across autoimmune pathologies. These cells dynamically modulate inflammatory cascades through diverse mechanisms (36–39). In psoriasis, SFRP2+ fibroblasts amplify the immune network via production of CCL13, CCL19 and CXCL12 (40). SFRP2^hi^PRSS23+WIF1- fibroblasts amplify immune response with a transcription into myofibroblasts in systemic sclerosis (19). In discoid lupus erythematosus (DLE), the inflammatory fibroblasts have been reported to originate from SFRP2+ fibroblast via the activation of interferon regulatory factors (IRF7, IRF1) and STAT1 (41). It was also reported that the reduction of Wnt5a+ fibroblasts is an early change in the resolution of skin inflammation after systemic or topical treatment in patients with psoriasis (42). In the future, specific surface molecules targeting disease-associated fibroblasts may become new therapeutic targets. Surprisingly, our study indicated that SFRP2+Wnt5a+ fibroblasts promoted the transition to an inflammatory state through the upregulation of interferon regulatory factors (IRF7, IRF8), STAT1 and STAT2. Consistently, the increased expression and colocalization of SFRP2 and Wnt5a were confirmed in primary cultured fibroblasts in NEOLP and EOLP *in vitro*, with the EOLP group exhibiting higher levels of SFRP2 and Wnt5a. Interferons (IFNs) have previously been reported to contribute to the transition of SFRP2+ fibroblasts to myofibroblasts in fibrosis, particularly in conditions like scleroderma (43, 44). Notably, our results showed that cytotoxic CD8+ T cells and exhausted CD8+ T cells produce IFNG, which may enhance the immune network by binding to receptors (IFNGR1, IFNGR2, F2R) on SFRP2+Wnt5a+ fibroblasts. Alternatively, we observed a close location of SFRP2+ fibroblasts and CD8+ T cells in OLP tissues. These findings highlight the important role of fibroblasts-CD8+ T cells crosstalk in shaping and maintaining the local immune environment in OLP. Evidence has shown that not only other myeloid cells, but also stromal cells such as fibroblasts, can acquire the capacity to cross-presentation, which may have significant impact on autoimmunity (45). SFRP2+ fibroblasts were reported to overexpress ERAP2 and HLA-C in psoriasis. In addition, ERAP2-high expression was reported to display a unique major histocompatibility complex-bound peptidome generated from intracellular antigens (38, 46). Here, our data demonstrated that SFRP2+Wnt5a+ fibroblasts had high expression of ERAP2 and HLA-A, and were enriched in antigen processing and presenting pathways. ERAP2 is known to be an aminopeptidase involved in the pathophysiology of autoimmune diseases, and the ERAP2/ERAP1 ratio results has been linked to increased autoimmunity risk. In addition, Wnt5a was reported to support antigen processing and activate CD8+ T cells (47). These results suggest that SFRP2+Wnt5a+ fibroblasts may mediate the formation of immune response disorders through antigen processing and presentation pathways. This provides new insights into the potential mechanisms by which fibroblasts mediate immune responses in OLP.

Exogenous or endogenous antigens have been reported to initiate the pathogenesis of OLP, though the specific antigen remains unclear (45). Studies have found that the KRT17 protein peptide in epithelial cells of psoriasis patients is associated with T cell proliferation and chemotaxis, suggesting it may act as an antigen to promote immune response (48, 49). As a skeletal component of epithelial cells, keratin (KRT) is known to maintain cell integrity and regulate their pro-inflammatory functions (50). The KRT17 protein may further promote T cell migration by activating chemokine formation in keratinocytes. Besides, in inflammatory skin disease, a keratin17-dependent mechanism amplifies neutrophil recruitment in stressed mouse skin (51). Notably, KRT17 protein was overexpressed in the epithelial layer of OLP, and enriched in antigen processing and presenting pathway, suggesting that KRT17 may amplify inflammatory responses as an antigen in oral lichen planus.

Keratinocyte apoptosis is the hallmark feature of pathological immune response in OLP (52). In order to better understand the detailed function of epithelial in differentiation states, we categorized epithelial cells into three differentiation states: basal, spinous, supraspinous. We further identified that SFRP2+Wnt5a+ fibroblasts communicated with the basal layer of epithelial cells via the production of the extracellular matrix protein tenascin C (TNC). TNC+ papillary fibroblasts have been identified as an inflammation-induced subset that promote psoriasiform skin inflammation, and TNC ablation in fibroblasts has been shown to alleviate skin inflammation in mice models of psoriasis (53). This suggests that SFRP2+Wnt5a+ fibroblasts may contribute to the chronic inflammation and risk of lesion recurrence in OLP through fibroblasts-epithelial cell crosstalk (54). Additionally, interferon alpha inducible protein 27 (IFI27) was differentially upregulated in epithelial layer of OLP, and the IFI27 expression was observed to rank firstly in the basal cell layer of EOLP, indicating that epithelial cells may be contribute to the IFN signaling pathway in their crosstalk with fibroblasts.

Our data provided a view of oral mucosa of OLP and we recognized limitations of our study. First, due to ethical constraints, it is difficult to obtain sufficient samples from normal persons that meet the quality inspection requirements. The sample size of our scRNA-seq data is limited, and the variations in patient age, sex and disease severity across our cohort may introduce confounding effects in the assessment of OLP’s inflammatory microenvironment. Second, one of the biggest challenges involved in OLP clinically is the long-term course and recurrence characteristics. We hypothesize that disease-specific subclusters promote this clinical feature, so we try to figure out the leading cellular contributor who promote the severity and the onset of OLP. Although the fibroblasts of EOLP group showed a higher expression of SFRP2 and Wnt5a compared to NEOLP group, the key driver of disease severity in OLP still needs further exploration. Third, some other cell types (such as B cells and NK cells) were also involved in the ligand-receptor interaction with fibroblasts in our data. In the future, we will expand the analysis of the interaction between fibroblasts and other immune cells, and conduct in-depth research on the molecular mechanisms of SFRP2+Wnt5a+ fibroblasts in OLP.

Taken together, our data provided a novel perspective on the role of SFRP2+Wnt5a+ fibroblasts in amplifying and maintaining local inflammation in OLP, and we also uncovered their communication network with CD8+ T cells and epithelial cells, shedding light on the mechanisms driving chronic inflammation. Our study expanded the understanding of the long-term course and relapsing characteristics of OLP in an in-depth sight of pathogenic fibroblasts.

## Data Availability

The datasets presented in this study can be found in online repositories. The names of the repository/repositories and accession number(s) can be found below: https://www.ncbi.nlm.nih.gov/, PRJNA1201624.
