# Supplementary material for: Single cell dissection reveals SFRP2+ fibroblasts amplifying inflammatory responses in oral lichen planus
Source: Front Immunol. 2025 Jun 12;16:1553963. doi: 10.3389/fimmu.2025.1553963 (PMC12197934; doi:10.3389/fimmu.2025.1553963)
Supplement: Supplementary file 1 [file Supplementaryfile1.docx]

**Supplementary data 1**

**Supplemental Materials and Methods**

**Table.S1** Clinical features of participants in scRNA-seq.

| Group | Participant ID | Age | Sex | REU score |
| --- | --- | --- | --- | --- |
| Non-erosive OLP | NEOLP1 | 29 | M | 3 |
|  | NEOLP2 | 28 | M | 3 |
| Erosive OLP | EOLP1 | 49 | F | 7 |
|  | EOLP2 | 54 | F | 15.5 |
| Healthy controls (HC) | HC | 18 | F | N/A |

REU score: Reticular, Erythema, and Ulceration score. F, Female; M, Male. N/A: not applicable.

**Table.S2** Clinical features of subjects in IHC.

| Group | NEOLP (n=20) | EOLP (n=23) | HC (n=18) |
| --- | --- | --- | --- |
| Age (Mean ± SEM) | 46.3 ± 2.77 | 50.04 ± 2.34 | 25.33 ± 1.05 |
| Sex (F/M) | 7/13 | 15/8 | 15/3 |

F, Female; M, Male.

**Table.S3** Clinical characteristics of OLP patients in qRT-PCR

| Group | NEOLP (n=25) | EOLP (n=26) | HC (n=24) |
| --- | --- | --- | --- |
| Age (Mean ± SEM) | 39.12 ± 2.13 | 48.76 ± 2.39 | 24.58 ± 1.18 |
| Sex (F/M) | 11/14 | 16/10 | 18/6 |

F, Female; M, Male.

**Table.S4 Antibody information**

| Antibody | Catalog | Dilution | Company | Applications |
| --- | --- | --- | --- | --- |
| SFRP2 | 66328-1-lg | 1:500 | Proteintech | IF |
| Wnt5a | 55184-1-AP | 1:200 | Proteintech | IF |
| Vimentin | bs-23063R | 1:200 | Bioss | IF |
| Cytokeratin | bsm-33062M | 1:300 | Bioss | IF |
| Cytokeratin 17 | 17516-1-AP | 1:500 | Proteintech | IHC |
| SFRP2 | 66328-1-lg | 1:100 | Proteintech | mIHC |
| Vimentin | bs-23063R | 1:200 | Bioss | mIHC |
| CD8a | 66868-1-lg | 1:1000 | Proteintech | mIHC |
| Wnt5a | 55184-1-AP | 1:200 | Proteintech | mIHC |
| Coralite488-conjugated Goat Anti-Rabbit IgG(H+L) | SA00013-2 | 1:200 | Proteintech | IF |
| Cy3-conjugated Affinipure Goat Anti-Mouse IgG(H+L) | SA00009-1 | 1:100 | Proteintech | IF |

**Table S5. Primers of qPCR**

| Genes | Forward (5’~3’) | Reverse (5’~3’) |
| --- | --- | --- |
| SFRP2 | CTGGCCCGACATGCTTGAG | GCTTCACATACCTTTGGAGCTT |
| Wnt5a | ATTCTTGGTGGTCGCTAGGTA | GGCCTTCTCCGATGTACTGC |
| HLA-A | GTTGAGAGCCTACCTGGATG | TGGTGGGTCATATGTGTCTTG |
| HLA-B | CAGTTCGTGAGGTTCGACAG | CAGCCGTACATGCTCTGGA |
| HLA-C | CGCCTACGACGGCAAGGATT | TGGTCAGAGAGGGGGTGGTG |
| ERAP2 | CCAGAGAAACTTACGCCTCAC | GCCTGGGTTGGCTCAAAATC |
| CXCL12 | ATTCTCAACACTCCAAACTGTGC | ACTTTAGCTTCGGGTCAATGC |
| CXCL14 | CGCTACAGCGACGTGAAGAA | GTTCCAGGCGTTGTACCAC |
| CCL2 | CAGCCAGATGCAATCAATGCC | TGGAATCCTGAACCCACTTCT |
| CCL19 | TACATCGTGAGGAACTTCCACT | CTGGATGATGCGTTCTACCCA |
| TNC | GCCCCTGATGTTAAGGAGCTG | GGCCTCGAAGGTGACAGTT |
| APOE | GTTGCTGGTCACATTCCTGG | GCAGGTAATCCCAAAAGCGAC |
| ACTB | CATGTACGTTGCTATCCAGGC | CTCCTTAATGTCACGCACGAT |
