# Supplementary material for: Single cell dissection reveals SFRP2+ fibroblasts amplifying inflammatory responses in oral lichen planus
Source: Front Immunol. 2025 Jun 12;16:1553963. doi: 10.3389/fimmu.2025.1553963 (PMC12197934; doi:10.3389/fimmu.2025.1553963)
Supplement: Supplementary file 2 [file Supplementaryfile2.pdf]

**Supplementary data 2**

**Supplemental Figures Legends**

**Figure.S1** A. UMAP plot showing the cells colored by disease conditions. B. UMAP plot showing the T cells colored by sub-clusters. C. UMAP plot showing the T cells colored by sub-clusters across the disease conditions. D-E. Dot plot of KEGG enrichment analysis of DEGs between NEOLP and HC, EOLP and HC in cytotoxic CD8+ T cells. F. Dot plot of KEGG enrichment analysis of DEGs between NEOLP and EOLP in exhausted CD8+ T cells.

**Figure.S2** A. Heatmap showing the representative marker genes for 5 sub-populations of fibroblasts in panel. B. Bar plot showing the different composition across by disease conditions for 5 sub-populations. C. Dot plot showing relative expression of selected genes signatures from published tissue pathogenic fibroblasts populations in fibroblasts of our data by disease conditions.

**Figure.S3** A. Primary cell culture of mucosal fibroblasts. B. Immunofluorescence for vimentin and cytokeratin in primary cultured fibroblasts. Vimentin (+), Cytokeratin (-).

**Figure.S4** A-K. mRNA expression of primary cultured fibroblasts in individuals (HC, n=24; NEOLP, n=25; EOLP, n=26) were measured by RT-qPCR. $*p< 0.05$, $**p< 0.01$, $***p< 0.001$, $****p< 0.0001$, ns, not significant.

**Figure.S5** A. Heatmap showing the representative marker genes for 5 sub-populations of SFRP2+ fibroblasts in panel. B. Contribution of each L-R pair with Wnt5a in SFRP2+ fibroblasts.

**Figure.S6** A-B. Dot plot showing antigen presenting associated expression of SFRP2+ fibroblasts between EOLP and HC, NEOLP and HC.

**Figure.S7** A-C. Heatmap showing the representative marker genes for 3 sub-populations of epithelial cells in panel. D. Dot plot of KEGG enrichment analysis of top20 DEGs between NEOLP and HC, EOLP and HC in basal layer of epithelial cells. E-F. Dot plot of KEGG enrichment analysis of top20 up-regulated DEGs between NEOLP and HC, EOLP and HC in for spinous layer (E) and supraspinous layer (F). G. The KRT17 positive value of IHC in individuals of HC, NEOLP and EOLP. $**p< 0.01$, $****p< 0.0001$.

**Figure.S8** A. Histogram showing the interaction strength of all cell types in EOLP, NEOLP and HC. B. Heatmap showing the differential interaction strength of all cell types. C. Violin plot showing the interaction strength of CXCL signaling genes in all

cell types. D. Histogram showing the contribution of each L-R pair in the MHC-I signaling interaction between the EOLP, NEOLP and HC.

**Figure.S9** A-D. Heatmap showing the differential signaling interaction strength between the EOLP, NEOLP and HC. Histogram showing the contribution of each L-R pair in the differential signaling interaction between the EOLP, NEOLP and HC.
