# Supplementary figures and images for: Single cell dissection reveals SFRP2+ fibroblasts amplifying inflammatory responses in oral lichen planus
Source: Front Immunol. 2025 Jun 12;16:1553963. doi: 10.3389/fimmu.2025.1553963 (PMC12197934; doi:10.3389/fimmu.2025.1553963)

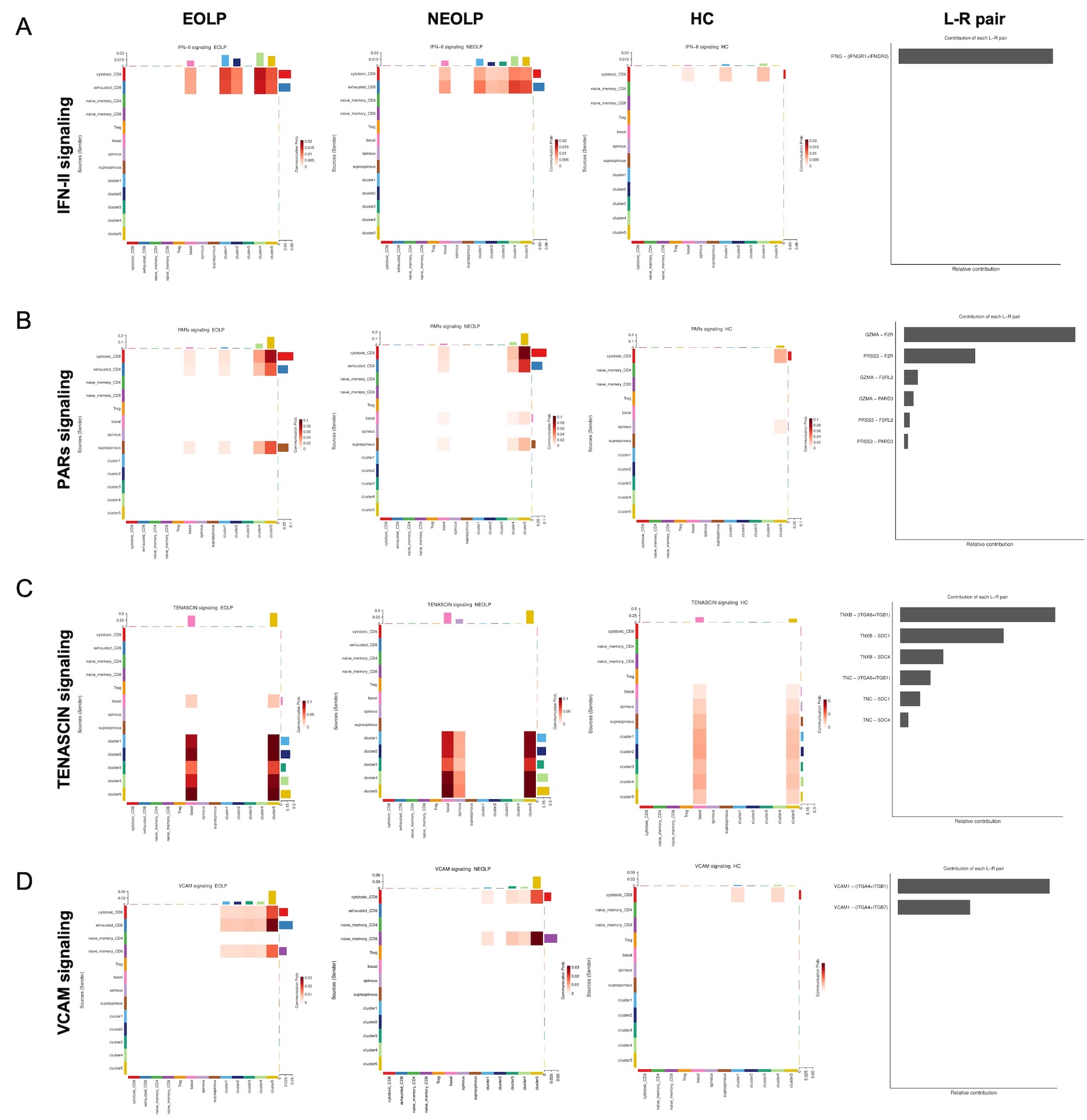

Supplement: Supplementary file 4 [file Supplementaryfile4.zip › Presentation 1/Supplementary figure9.jpg]

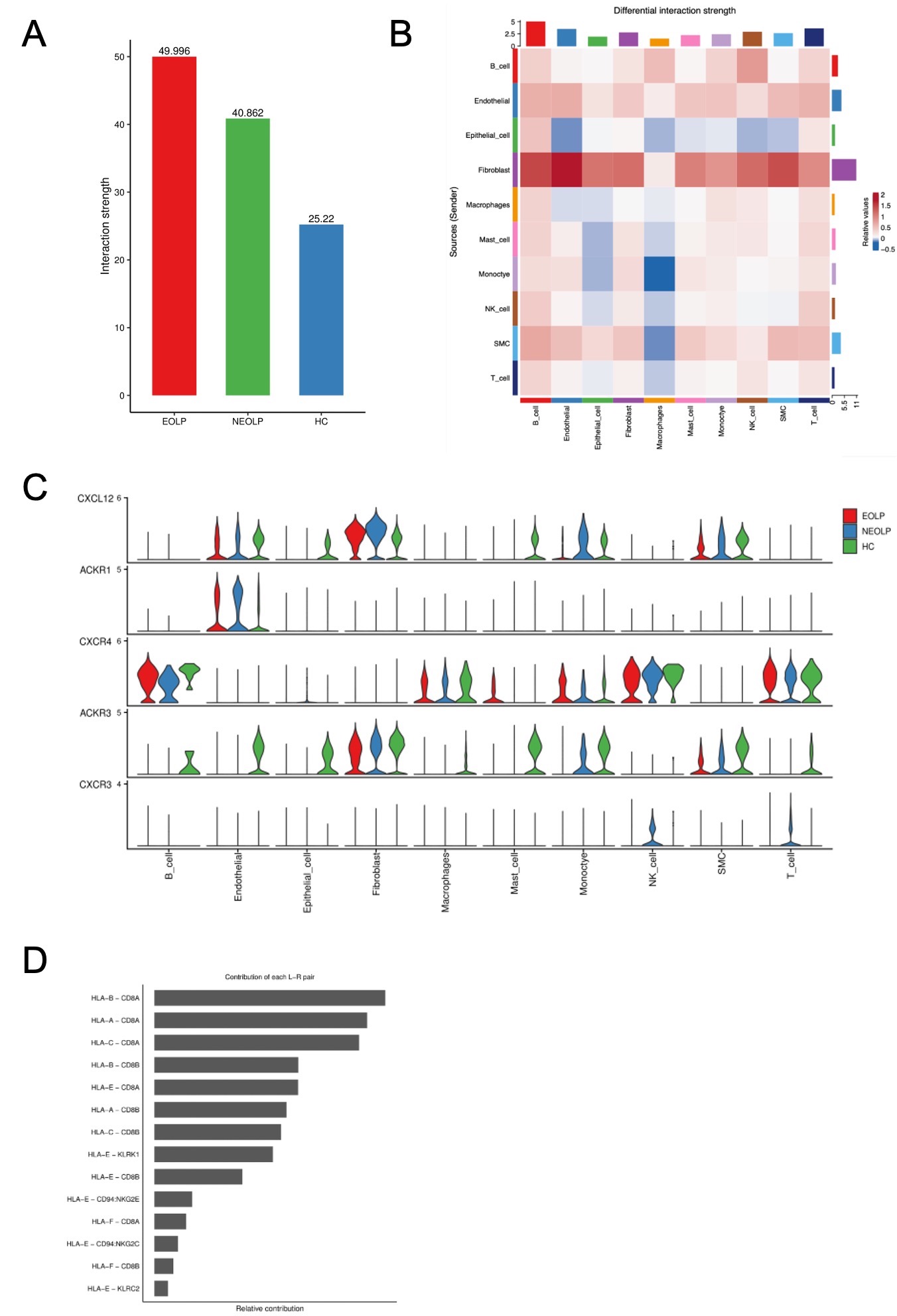

Supplement: Supplementary file 4 [file Supplementaryfile4.zip › Presentation 1/Supplementary figure8.jpg]

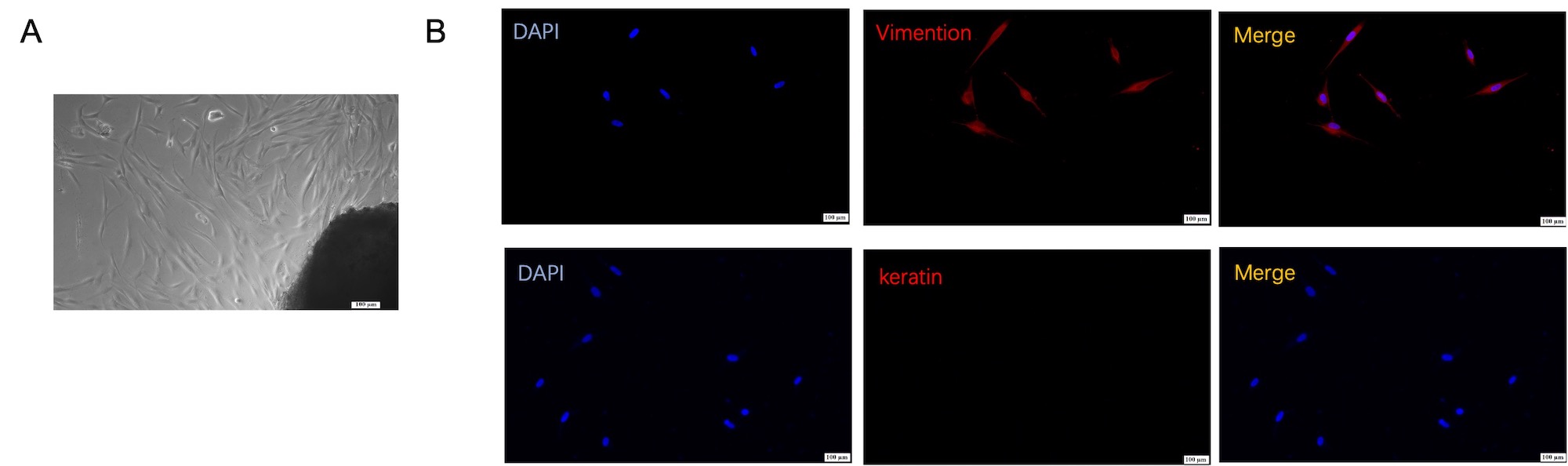

Supplement: Supplementary file 4 [file Supplementaryfile4.zip › Presentation 1/Supplementary figure3.jpg]

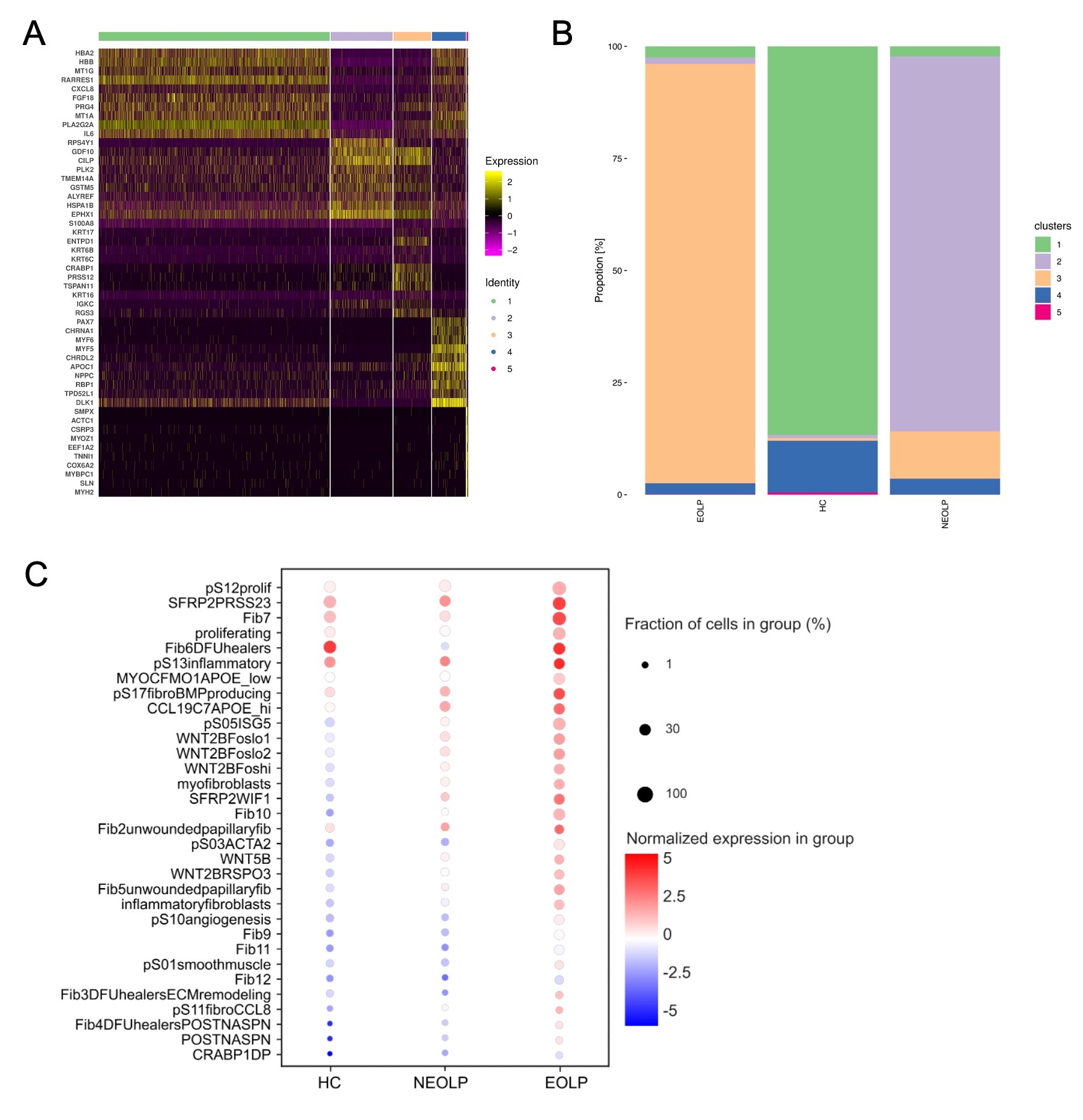

Supplement: Supplementary file 4 [file Supplementaryfile4.zip › Presentation 1/Supplementary figure2.jpg]

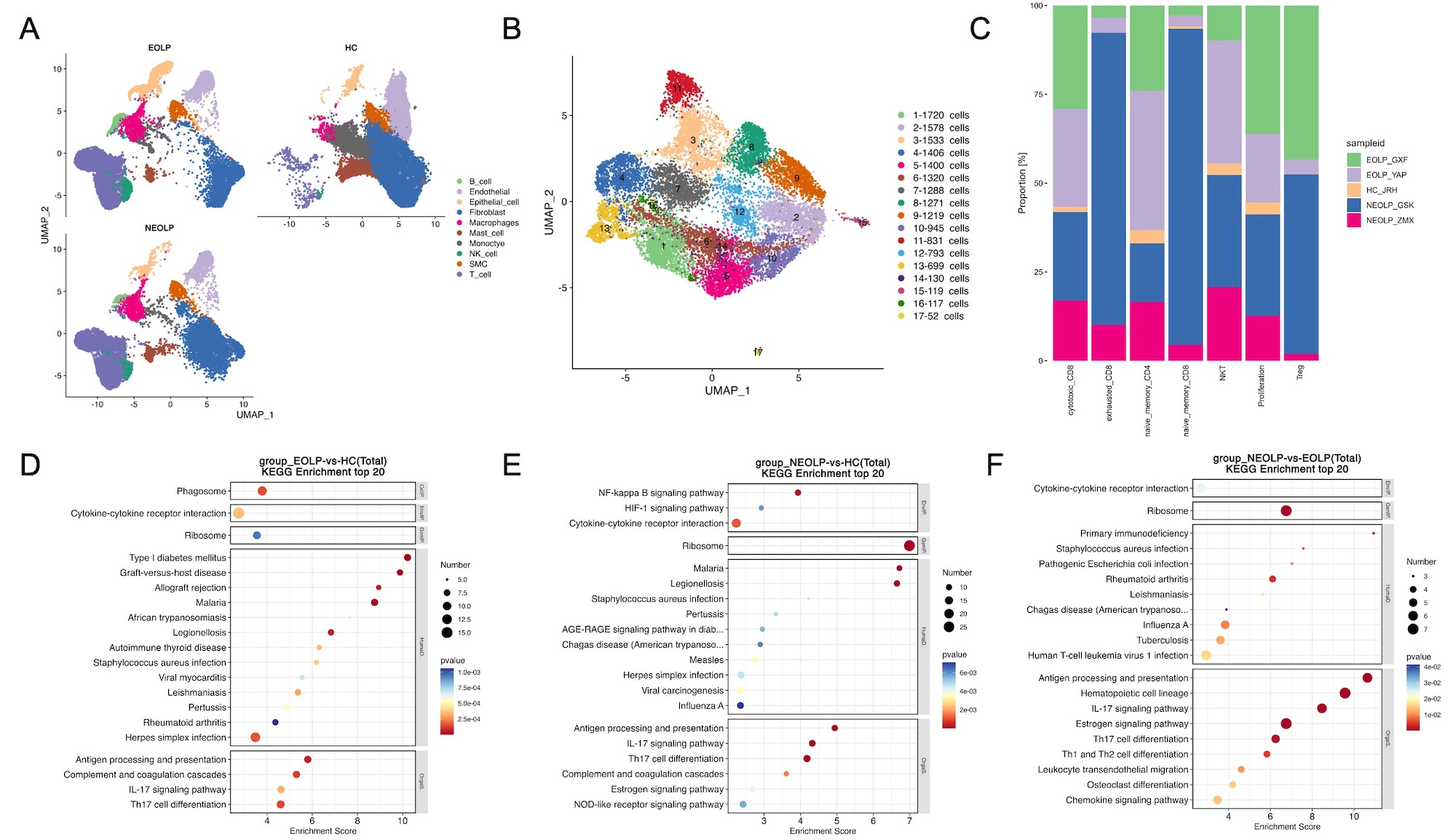

Supplement: Supplementary file 4 [file Supplementaryfile4.zip › Presentation 1/Supplementary figure1.jpg]

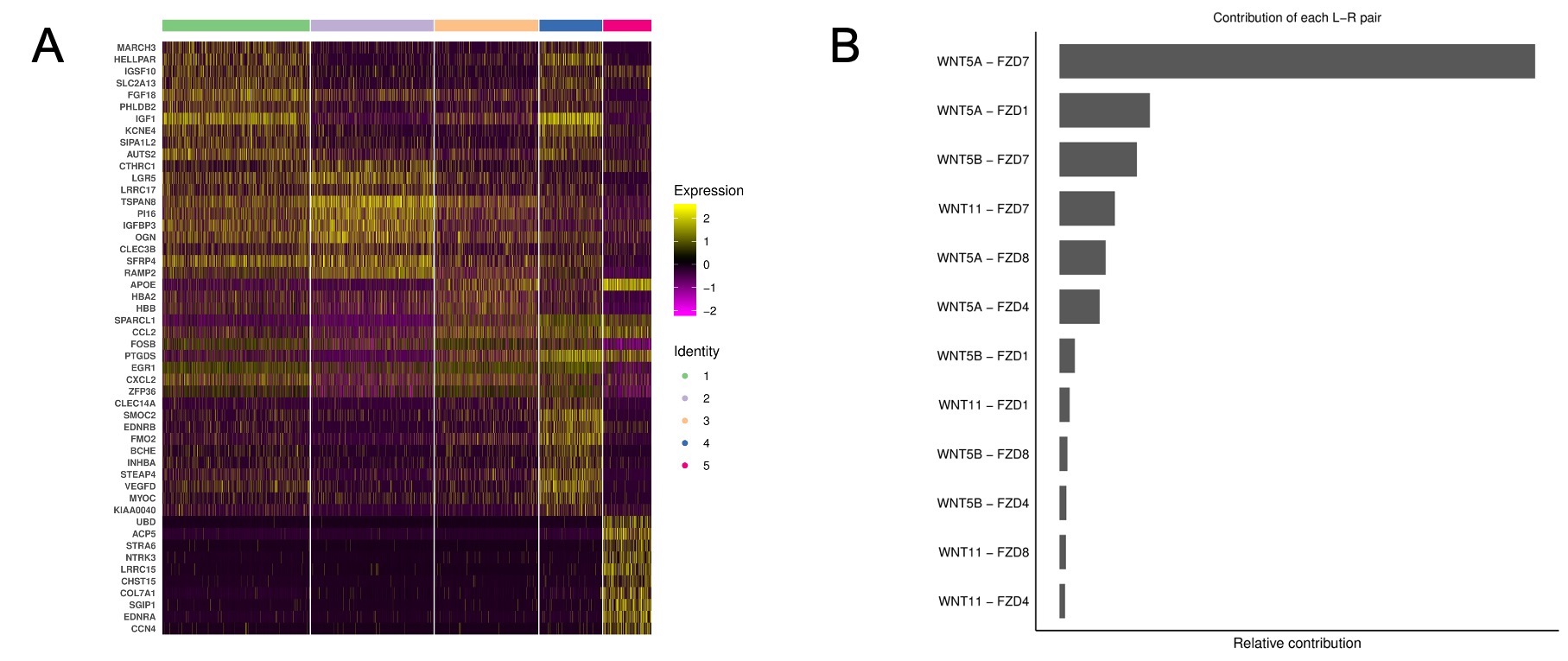

Supplement: Supplementary file 4 [file Supplementaryfile4.zip › Presentation 1/Supplementary figure5.jpg]

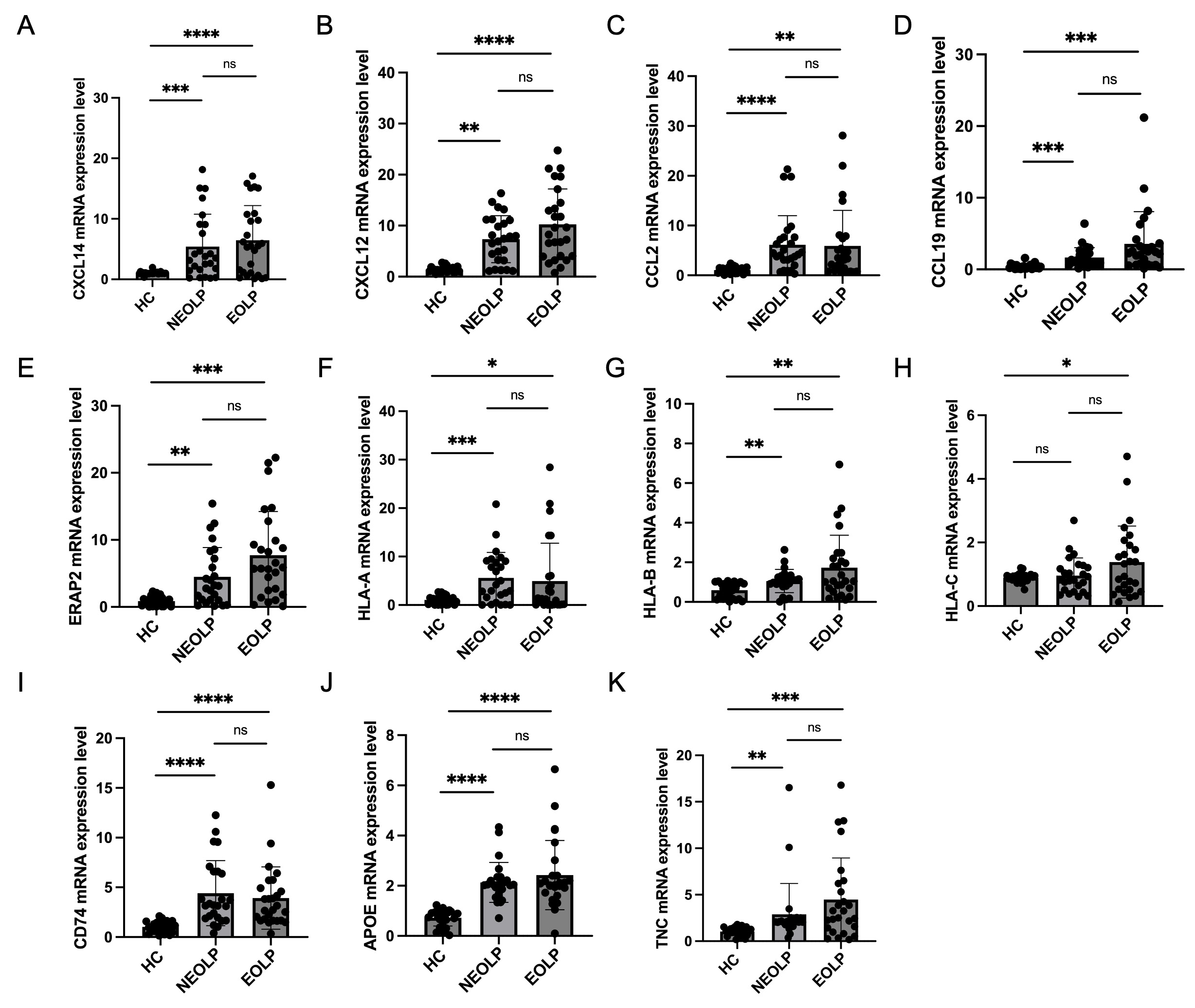

Supplement: Supplementary file 4 [file Supplementaryfile4.zip › Presentation 1/Supplementary figure4.jpg]

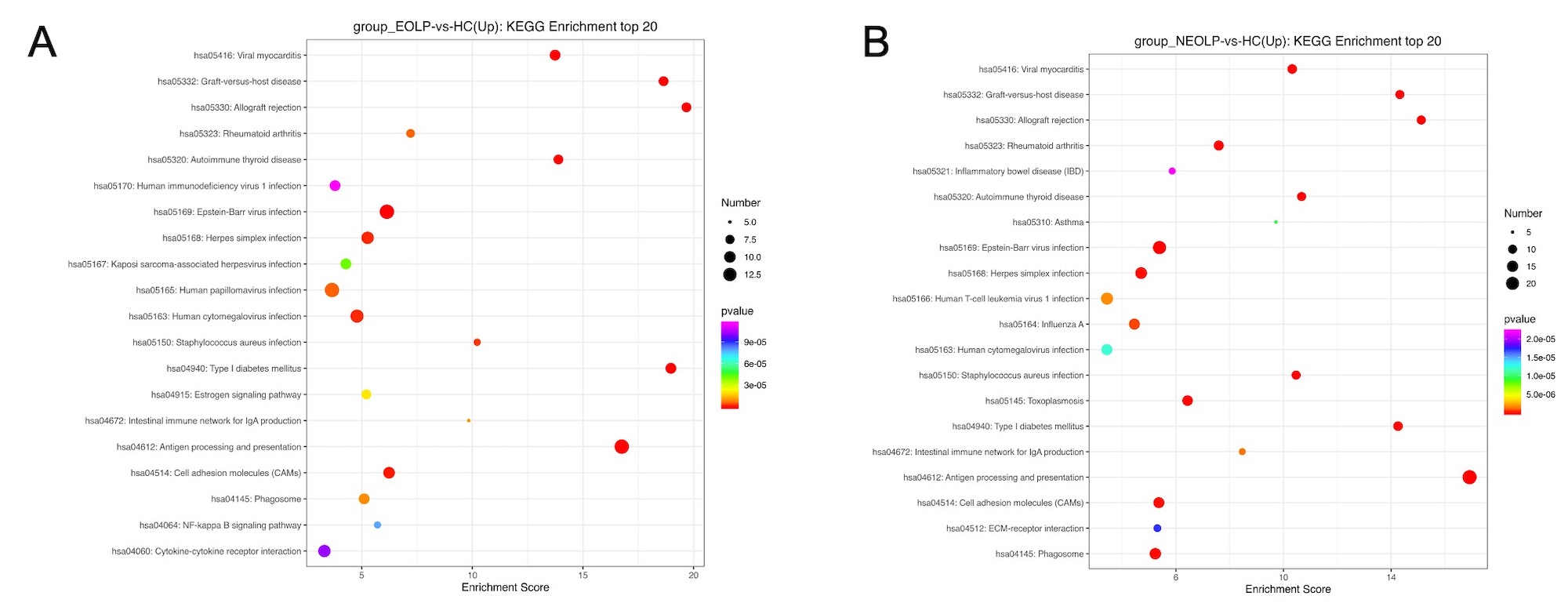

Supplement: Supplementary file 4 [file Supplementaryfile4.zip › Presentation 1/Supplementary figure6.jpg]

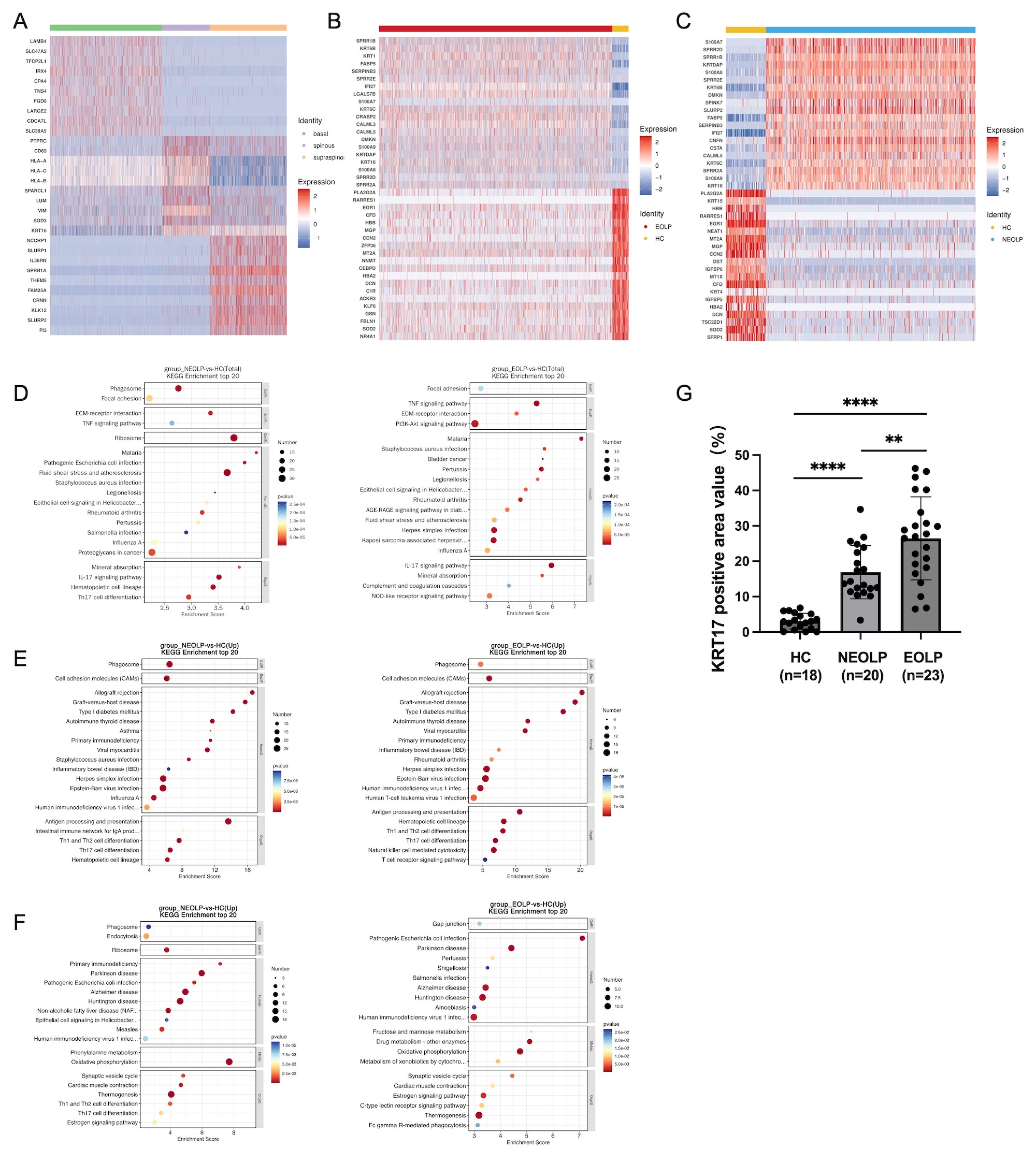

Supplement: Supplementary file 4 [file Supplementaryfile4.zip › Presentation 1/Supplementary figure7.jpg]
